# Prevalence and impact of comorbid obstructive sleep apnoea in diffuse parenchymal lung diseases

**DOI:** 10.1371/journal.pone.0246878

**Published:** 2021-02-11

**Authors:** Diandian Li, Bo Wang, Yi Liu, Haohua Wang

**Affiliations:** 1 Department of Respiratory and Critical Care Medicine, West China Hospital of Sichuan University, Chengdu, Sichuan, China; 2 West China School of Medicine, Sichuan University, Chengdu, Sichuan, China; University of Milano Biococca, ITALY

## Abstract

**Objective:**

Obstructive sleep apnea (OSA) are increasingly recognized as important features in diffuse parenchymal lung diseases (DPLDs) with differed prevalence and impact reported. The aim of this study is to systematically review the prevalence of comorbid OSA and characterize its impact on clinical and outcome measurements in adults with DPLDs.

**Methods:**

Publications addressing the prevalence of OSA in DPLDs and its impacts on DPLDs were selected from electronic databases. A random-effect model was used to estimate the pooled prevalence of OSA. Odds ratios (ORs) or mean differences (MDs) were used to assess the associations of OSA with clinical and outcome measurements. Heterogeneity was quantified by I^2^ with 95% confidence interval (95% CI).

**Results:**

4 studies comprising 643 participants were included. Overall, the pooled prevalence of OSA among DPLDs was 72% (95% CI: 65–79%; I^2^ = 75.4%). Moderate-severe OSA was observed in 40% patients (95% CI: 28–52%; I^2^ = 90.8%). The prevalence was higher as 76% in idiopathic pulmonary fibrosis (IPF) patients than in connective tissue associated-ILD or sarcoidosis (60%). Although oxygen desaturation during sleep was greater in OSA group compared with non-OSA patients, there was no difference in lung function or systematic comorbidities between the two groups. The associations between OSA and the mortality or disease progression of DPLDs were also systematically reviewed.

**Conclusion:**

In conclusion, OSA is a common comorbidity in DPLD patients, affecting approximately three in four patients, which may exacerbate the nocturnal desaturation and have negative influence on the outcomes. Larger studies with more homogeneous samples are warranted.

## Introduction

Diffuse parenchymal lung diseases (DPLDs), also known as interstitial lung diseases (ILDs), are a heterogeneous group of disorders characterized by varying degrees of inflammation and fibrosis resulting in damage to the lung parenchyma and derangement of the gas-exchange units of the lung [1,2]. Fibrotic DPLDs like idiopathic pulmonary fibrosis (IPF) often relate to restrictive ventilatory abnormalities and impaired gas exchange that may progress to respiratory failure, poor quality of life with a 5-year survival rate from 20% to 40% [3].

Sleep represents a state of restoration in normal people, covering approximately one third of human life [4]. Obstructive sleep apnea (OSA), a common type of sleep breathing disorders (SBD), is characterized by recurrent upper airway obstruction and is recognized as a significant health problem with increasing prevalence, affecting approximately 20–30% of the adult population [5,6]. In 2007, Mermigkis et al. reported the need for a high awareness for OSA in IPF population, which confirmed OSA in 11 out of 18 IPF patients. Afterwards, the most recent IPF guidelines in 2011 include for the first time OSA in IPF-associated comorbidities [7]. Since then, an increasing number of small-sample size studies have focused on the role of OSA in IPF and other DPLDs, with differed prevalence and impact reported. Previous physiological studies have shown that the mechanism underlying OSA and fibrotic ILDs linkage might be correlated with the decreased lung volumes. In these diseases, reduced upper airway stability and increased resistance facilitate upper airway collapse, especially during rapid eye movement (REM) sleep period when the functional residual capacity is further reduced due to intercostal muscle inactivity [8,9]. Based on the abovementioned theories, some studies suggested that underlying OSA appeared to have a negative influence on morbidity and mortality in DPLD patients, and should be timely diagnosed and treated [10,11].

However, to date, few studies have systematically and comprehensively evaluated the epidemiology of OSA as a comorbidity and its impact on DPLDs. Some studies highlighted the potential importance of OSA with prevalence estimates of up to 90% patients with IPF [10,12,13], while others showed a much lower prevalence of OSA of 45% in IPF patients [14]. Also, it remains unclear if this phenomenon is pathogenic or simply indicative of advanced disease. Most individual cohort studies included a few patients with advanced lung disease, and the sample size for some studies was small. Differences in study design and population characteristics may confound a true understanding and appreciation of the relevance of comorbid OSA in the disease.

As meta-analysis is an important tool to reliably and accurately summarize the current evidence, the aim of this study is to systematically review the prevalence of comorbid OSA and characterize its impact on clinical and outcome measurements such as oxygenation, lung function, sleep quality, comorbid conditions and survival in adults with DPLDs.

## Methods

### Search strategy

A systematic literature search was performed to identify studies which included information addressing the following research questions: (a) What is the prevalence of OSA in adult patients with DPLDs? (b) How does comorbid OSA impact on clinical and outcome measurements of DPLDs, such as oxygenation, lung function, sleep quality, comorbid conditions or survival?.

An electronic search of PubMed, Web of Science, Embase, and Cochrane Library (updated to January 1, 2020) was conducted by two investigators (DL and YL). The syntax used for search was ((diffuse parenchymal lung disease) OR (DPLD)) OR (interstitial lung disease)) OR (ILD)) OR (idiopathic pulmonary fibrosis)) OR (IPF)) OR (sarcoidosis)) AND ((OSA) OR (SHS) OR (OSAHS) OR (SAHS) OR (hypopnea) OR (hypopnoea) OR (obstructive sleep apnea) OR (sleep breathing disorders)). Language was restricted to English. We also searched the reference lists in the initially identified articles to get additional relevant records. Disagreements were resolved at each step by consensus. This study was performed according to the recommendations of the Preferred Reporting Items for Systematic Reviews and Meta-Analyses (PRISMA) statement [15].

### Selection criteria

Studies were included if they met the following criteria: (a) studies specified the diagnostic criteria of DPLDs and objectively defined OSA, and (b) the prevalence or clinical relevance of OSA in DPLDs had to be reported, and (c) age ≥ 18 years. Studies were excluded if: (a) the diagnosis of OSA was only based on questionnaires or clinical symptoms, or (b) the prevalence of OSA in DPLDs were not reported, or (c) no original data were present, or (d) duplicate data. Reviews, letter to editor or comments conference abstracts were excluded due to insufficient data. Any discrepancy was resolved by discussion between the authors.

### Endpoints studied

The primary endpoint was the pooled prevalence of OSA in DPLDs. Secondary endpoints included: (a) the pooled mean differences (MDs) in sleep stage, arousal index, lung function parameters (% predicted FEV1, FVC, DLCO and TLC), 6-min walk test (6MWD), oxygen desaturation during sleep (oxygen desaturation index (ODI) and T_90_), arterial blood gas variables between DPLD patients with OSA and without OSA; (b) the pooled odds ratios (ORs) of the prevalence of comorbidities between DPLD patients with and without OSA; (c) mortality and disease progression between DPLD patients with and without OSA.

### Data extraction

Two investigators (DL and YL) independently extracted data, blinded to the authors and institutions of the included studies. Discrepancies (if any) were addressed by joint re-evaluation of the original article. The following information was extracted from each study: first author, publication year, region, study design, data source, population characteristics, diagnostic criteria, polysomnographic and physiologic parameters, outcomes, and prevalence of OSA.

Since there is no widely accepted tool to assess the quality of patient-based prevalence studies, we referred to the assessment tools from a previous meta-analysis [16], which included sample size justification (consecutive or random sample), appropriate selection criteria, case definition (an objective case definition), and outcome definition (the study instrument for the measurement of OSA sufficiently specific). Two investigators (DL and HW) independently scored the quality of the studies using the same scale. Disagreements between the reviewers were resolved by consensus with a third author (BW).

### Statistical analysis

Random-effect meta-analyses were used to estimate the pooled prevalence of OSA due to potential clinical and methodological heterogeneities in observational studies [17]. The discontinuous parameters were compared by ORs with 95% confidence intervals (95% CIs) using Mantel Haenszel (MH) method, while MDs with 95% CIs were selected to compare the continuous parameters that were reported in identical scales across all studies. A chi-squared test was used to quantify heterogeneity by means of an I^2^ with 95%CI. Sensitivity analyses were also performed to further explore the heterogeneity according to study characteristics. The publication bias was estimated using the Eggers test when appropriate. A P-value <0.05 was considered as statistically significant. Analyses were carried out using Stata version 12.0 (Stata Corporation, College Station, TX) and Revman5.3 software (Cochrane Collaboration, Oxford, UK).

## Results

### Characteristics of eligible studies

**Fig 1:**
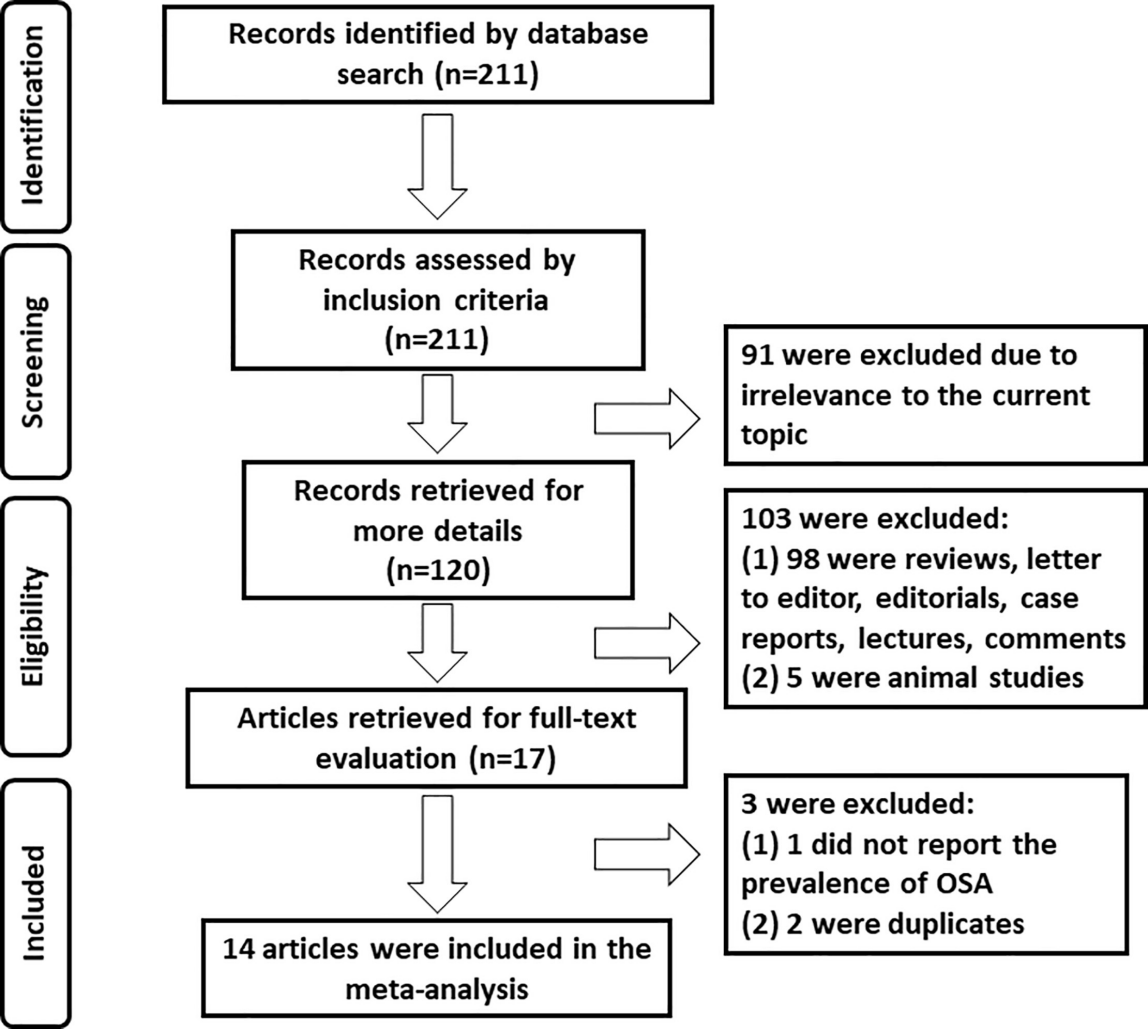
Flow chart of selection process for eligible articles.

From the 211 studies initially retrieved publications, a total of 14 studies comprising 643 participants were finally selected. A flow chart outlining the selection process and detailed identification is presented in **Fig 1**. These studies were published between 2007 and 2019. All but one study [18] collected data prospectively. The number of study participants ranged from 18 to 92. The characteristics of these 14 studies included in the meta-analysis are presented in **Table 1**. Quality assessment for each study is summarized in **S1 Table**.

**Table 1:** Summary of eligible studies.

First author	Year	Country	Study design	Data source	Study subjects	Sample size	Average age (years) (mean ± SD)	Male (%)	BMI	Diagnostic tool	OSA (n)
Mermigkis C [22]	2007	Greece	prospective	single-center	IPF	18	68.1±8.8	67	33.2±6.9	PSG	11
Lancaster LH [13]	2009	USA	prospective	single-center	IPF	50	64.5±8.5	66	32.2±5.9	PSG	44
Mermigkis C [23]	2010	Greece	prospective	multi-center	IPF	34	65±10.6	62	27.3±4	PSG	20
Pihtili A [26]	2013	Turkey	prospective	single-center	IPF/II-III sarcoidosis/scleroderma	50	53.92±12.19	28	25.94±2.32	PSG	34
Kolilekas L [10]	2013	Greece	prospective	single-center	IPF	31	67.96±7.88	77	28.66±4.3	PSG	28
Lee RN [14]	2015	Ireland	prospective	single-center	IPF	20	67.9±12.3	NA	28.50±4.56	PSG	9
Mavroudi M [21]	2017	Greece	prospective	single-center	IPF/II-III sarcoidosis	40	61.4±9.36	45	28.89±5.14	PSG	27
Bosi [19]	2017	Italy	prospective	single-center	IPF	35	68.23±9.48	77	22.8±3.6	PSG	25
Gille T [12]	2017	France	prospective	multi-center	IPF	45	68.8±8.7	84	28±3.5	PSG	40
Cardoso AV [20]	2018	Portugal	prospective	single-center	fibrotic ILD	49	67.2±12.2	53	NA	Overnight cardiorespiratory polygraphy	34
Zhang XL [27]	2019	China	prospective	single-center	fibrotic ILD	77	65.1±9.4	74	25.2±3.3	PSG	48
Troy LK [24]	2019	Australia	prospective	single-center	ILD	92	66.1±10.7	55	30.7±5.7	PSG	60
Tudorache V [18]	2019	Romania	retrospective	single-center	IPF	23	67.6±8.7	57	27.7±4.5	Overnight cardiorespiratory polygraphy	19
Sarac S [25]	2019	Turkey	prospective	single-center	IPF/sarcoidosis/NSIP/CTD-ILD/BOOP	79	55.4±11.3	51.9	28.7±4.7	PSG	53

BOOP, bronchiolitis obliterans organized pneumonia; CTD, connective tissue disease; IPF, idiopathic pulmonary fibrosis; ILD, interstitial lung diseases; NSIP, non-specific interstitial pneumonia;OSA, obstructive sleep apnoea; PSG, polysomnography.

### Prevalence of OSA in DPLDs

**Fig 2:**
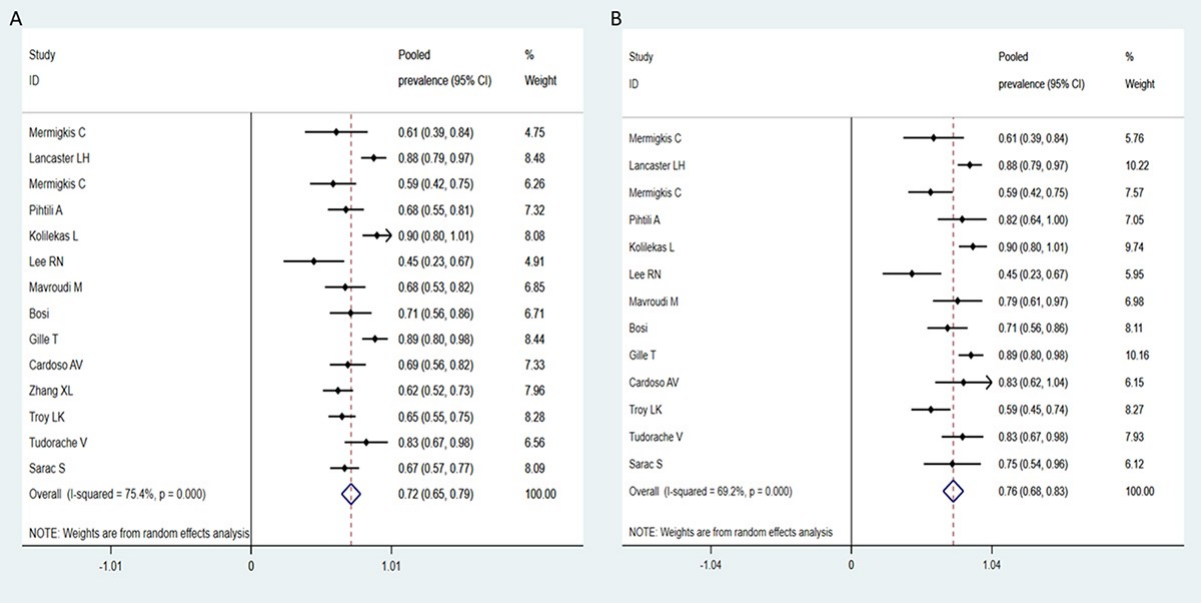
Funnel plot of the prevalence of OSA in DPLD patients (A) and in IPF patients (B).

Overall, the pooled prevalence of OSA among DPLDs was 72% (95% CI: 65–79%; I^2^ = 75.4%), ranging from 45 to 90% (**Fig 2A**). Based on 12 studies including 546 patients, which reported severity of OSA [10,12,13,18–26], moderate–severe OSA was observed in 40% patients (95% CI: 28–52%; I^2^ = 90.8%), ranging from 8 to 68%, and the pooled prevalence of mild OSA was equally high as 36% (95% CI: 23–48%; I^2^ = 91.5%), ranging from 6 to 67%.

Subgroup analyses were performed to evaluate sources of heterogeneity in the prevalence estimates between studies. The results revealed the prevalence to be higher as 76% (95% CI: 68–83%; I^2^ = 69.2%) in the 13 studies enrolling IPF patients [10,12–14,18–26] (**Fig 2B**), whereas in 4 studies including patients with connective tissue associated-ILD or sarcoidosis [20,21,25,26], the pooled prevalence of OSA was much lower (60%, 95% CI: 49–70%; I^2^ = 0%), suggesting that the prevalence differed according to the type of DPLDs. Moreover, the prevalence of comorbid OSA in healthy controls and chronic obstructive pulmonary disease (COPD) were examined in two studies, and were found to be less frequent than in IPF (AHI 1.7 ± 1.0 vs 11.6 ± 7.2 [21]; prevalence 44% vs 62% [27]). The possible effect of different hypopnea scoring criteria was also examined. Although the number of included studies per subgroup was small and the heterogeneity was large, the pooled prevalence did not greatly differ according to hypopnea scoring criteria employed (**S2 Table**).

The prevalence also differed based on the population characteristics. As expected, the five studies that recruited patients with average BMI less than 28 revealed a lower prevalence of OSA (68%, 95% CI: 61–76%; I^2^ = 34.8%) than that of studies enrolling patients with average BMI more than 28 (73%, 95% CI: 64–82%; I^2^ = 65.2%).

**Fig 3:**
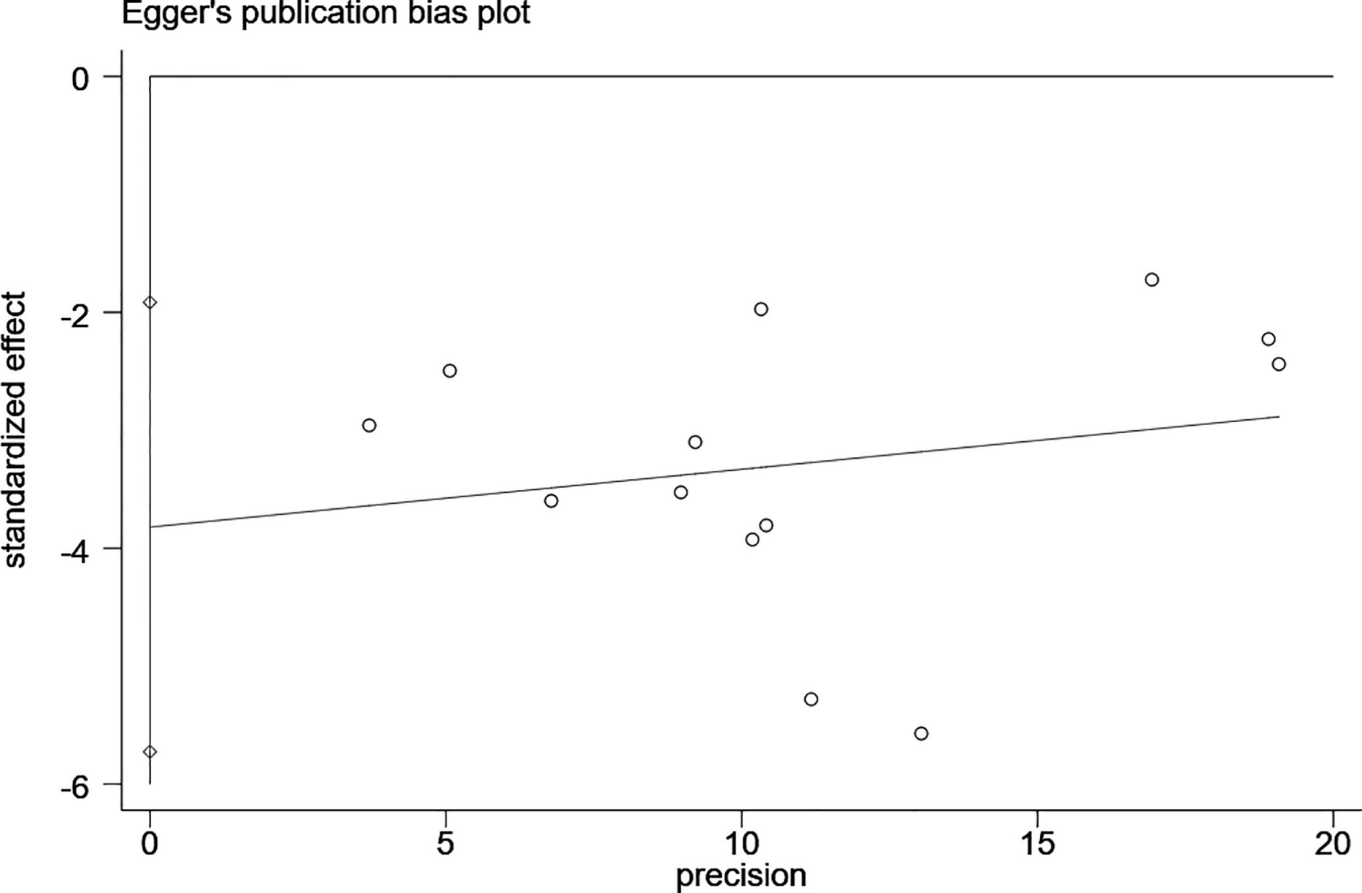
Egger’s plot to detect publication bias on overall estimate.

In sensitivity analyses, it was found that study design, sample representativeness, lack of patient inclusion/exclusion criteria or specific criteria to define DPLDs, or publication year were not a significant source of heterogeneity in the prevalence estimates (**Table 2**). Publication bias in current meta-analysis was tested by Egger’s test with P = 0.001 (**Fig 3**).

**Table 2:** Meta-analysis of the overall prevalence of OSA in DPLDs.

Variable	Number of studies	Number of patients	Prevalence of OSA (95% CI)	I^2^ (%)
Total	14	643	0.72 (0.65–0.79)	75.4
Study design				
Prospective	13 [10,12–14,19–27]	620	0.71(0.64–0.78)	76.8
Retrospective	1 [18]	23	0.83(0.67, 0.98)	-
Sample representativeness				
Single-center	12 [10,13,14,18–22,24–27]	564	0.71(0.64–0.78)	71.9
Multi-center	2 [12,23]	79	0.75(0.45–1.04)	89.7
Description of inclusion criteria				
No	7 [10,18,19,22–24,27]	310	0.71(0.61–0.81)	72.7
Yes	7 [12–14,20,21,25,26]	333	0.72(0.62–0.82)	79.1
Description of exclusion criteria				
No	4 [10,13,22,23]	128	0.77(0.62–0.92)	79.9
Yes	10 [12,14,18–21,24–27]	515	0.70(0.63–0.77)	66.7
Specific criteria to define DPLD				
No	1 [20]	49	0.69(0.57–0.82)	-
Yes	13 [10,12–14,18,19,21–27]	594	0.72(0.65–0.79)	77
Publication year				
Before 2015	5 [10,13,22,23,26]	183	0.74(0.63–0.85)	76.6
After 2015	9 [12,14,18–21,24,25,27]	460	0.70(0.61–0.79)	73.8

DPLD, diffuse parenchymal lung diseases; OSA, obstructive sleep apnoea; CI, confidence interval.

### OSA and DPLD outcomes

As shown in **Table 3**, several studies reported associations of OSA with physiologic and polysomnographic parameters. Although DPLD patients combined with OSA had more N1 and N2 but less N3 and REM sleep, the overall sleep distribution was similar between patients with and without OSA. In addition, arousal index was higher in OSA group. As expected, oxygen desaturation during sleep was greater in OSA group (as reflected by ODI and T_90_) in comparison with non-OSA patients. However, the presence of OSA was not significantly associated with 6MWD, FVC, TLC, FEV1, DLCO or arterial blood gas variables.

**Table 3:** Comparison of physiological and polysomnographic parameters between DPLD patients with and without OSA.

Variable	Number of studies	Number of patients		MD (95% CI)	*P*-value	I^2^ (%)	*P*_*het*_
		OSA	Non-OSA				
FEV1 (% predicted)	5 [12,14,20,23,27]	151	74	3.88 (-2.78, 10.55)	0.25	31	0.22
FVC (% predicted)	6 [12–14,20,23,27]	195	80	5.00 (-0.84, 10.83)	0.09	40	0.14
DLCO (% predicted)	6 [12–14,20,23,27]	195	80	0.05 (-4.95, 5.06)	0.98	0	0.62
TLC (% predicted)	5 [12,14,20,23,27]	151	74	-0.98 (-6.88, 4.93)	0.75	48	0.11
6MWD (meters)	2 [19,20]	59	24	-14.16 (-77.17, 48.85)	0.66	0	0.88
PaO2 (mmHg)	5 [12,14,19,20,27]	156	69	1.14 (-2.70, 4.99)	0.56	0	0.57
PaCO2 (mmHg)	5 [12,14,19,20,27]	156	69	0.88 (-0.94, 2.69)	0.34	17	0.31
T_90_ (%TST)	5 [12,19,20,23,27]	167	72	8.98 (5.54, 12.42)	<0.001	0	0.98
ODI (events/hr)	4 [12,13,19,27]	133	57	19.07 (12.98, 25.16)	<0.001	79	0.003
N1 (%TST)	5 [12–14,19,27]	166	60	4.01 (-1.77, 9.80)	0.17	68	0.01
N2 (%TST)	5 [12–14,19,27]	166	60	0.11 (-6.67, 6.89)	0.97	78	0.001
N3 (%TST)	5 [12–14,19,27]	166	60	-2.49 (-4.96, -0.02)	0.05	0	0.48
REM (%TST)	5 [12–14,19,27]	166	60	-0.37 (-2.71, 1.97)	0.76	5	0.38
Arousal index(events/hr)	4 [12–14,27]	141	51	14.93 (3.99, 25.87)	0.007	90	<0.001

FEV1, force expiratory volume in 1 second; FVC, forced vital capacity; DLCO, carbon monoxide diffusion capacity; TLC, total lung capacity; 6MWD, 6 minutes walking distance; PaO2, arterial oxygen partial pressure; PaCO2, arterial carbon dioxide partial pressure; TST, total sleep time; T _90_, percentage of total sleep time spent with SpO2<90%; ODI, oxygen desaturation index; N1, percentage of total sleep time spent in NREM sleep stage1; N2, percentage of total sleep time spent in NREM sleep stage 2; N3, percentage of total sleep time spent in NREM sleep stage3; REM percentage of total sleep time spent in REM sleep; OSA, obstructive sleep apnoea; MD, mean difference; CI, confidence interval; *P*_*het*_, *P*-value for heterogeneity.

As shown in **Table 4**, the pooled prevalence of gastro-esophageal reflux disease (GERD), hypertension, coronary disease, diabetes and stroke were 57%, 49%, 20%, 20% and 8%, respectively. The prevalence was not significantly different between patients with and without OSA. Besides, one study [20] reported that pulmonary hypertension was present in 18.2% OSA and 15.3% non-OSA patients, but in another study [13], no significant difference in the calculated systolic pulmonary artery pressure was found in subjects with and without OSA.

**Table 4:** Comparison of comorbidities between DPLD patients with and without OSA.

Variable	Number of studies	Number of patients (Events/Total)		OR (95% CI)	*P*-value	I^2^ (%)	*P*_*het*_
		OSA	Non-OSA				
Hypertension	4 [13,14,20,27]	25/61	71/135	0.86 (0.44, 1.68)	0.65	0	0.68
Diabetes mellitus	4 [13,14,20,27]	10/61	29/135	0.72 (0.32, 1.65)	0.44	0	0.91
Coronary artery disease	4 [13,14,20,27]	10/61	29/135	0.66 (0.29, 1.51)	0.32	0	0.57
GERD	3 [13,14,20]	16/32	52/87	1.32 (0.14, 12.27)	0.81	71	0.06
Stroke	2 [20,27]	4/44	6/82	1.30 (0.33, 5.07)	0.71	0	0.56

GERD, gastro-esophageal reflux disease; OSA, obstructive sleep apnoea; OR, odds ratio; CI, confidence interval; *P*_*het*_, *P*-value for heterogeneity.

Only three studies reported the associations between comorbid OSA and the prognosis of DPLDs, such as mortality and disease progression. Nevertheless, meta-analysis could not be conducted here due to a lack of commonality in assessment measurements. Kolilekas et al reported that apnea-hypopnea index (AHI) was significantly correlated with decreased survival of IPF patients not receiving treatment with continuous positive airway pressure (CPAP) (HR = 1.02, 95%CI: 1.001–1.048, P = 0.043) [10]. Another study identified the presence of OSA correlated with sleep-related hypoxemia as the only predictor of mortality (HR = 6.7, 95% CI 1.2–36.3, P = 0.029) and the presence of SDB a risk factor for disease progression in IPF (HR = 3.1, 95% IC 1.44–6.3, P = 0.003) [19]. However, in Troy’s study, moderate-severe OSA was not an independent predictor of progression-free survival in DPLDs (HR = 0.79, 95% IC 0.33–1.92, P = 0.61) [24].

## Discussion

In the present systematic review and meta-analyses, we summarized the results of 14 clinical studies representing 643 DPLD patients. The pooling results demonstrate that OSA is a common comorbidity in DPLD patients, affecting approximately three in four patients, and is more common in IPF population than in patients with CTD-ILDs or sarcoidosis. Moderate-severe OSA was observed in 40% DPLD patients. Notably, these high prevalence estimates also remained consistent in subgroup analyses of studies based on patients with average BMI less than 28, suggesting that obesity was not a major risk factor for OSA in DPLDs.

Debate remains as to the causality of OSA and DPLD. Previous data from the Sleep Heart Health Study (SHHS) have shown that in the 60–69-year age group, prevalence of mild and moderate-to-severe OSA of 32% and 19%, respectively, and 51% of people had an AHI above 5 events/hour [28]. As IPF is known to occur mostly in this age group, it might be questioned whether the high prevalence of OSA is truly associated with IPF. Two included studies compared the prevalence of OSA in IPF patients with that in non-ILD controls (healthy controls and COPD patients) [21,27], which showed that OSA were diagnosed less frequently in non-ILD controls. The pooled prevalence of OSA in patients with CTD-ILD or sarcoidosis was also lower than that in IPF. Notably, in these cohorts, there is a heterogeneity between IPF and non-IPF patients concerning the average age and gender, which may affect the frequency of OSA. However, compared with people in the age group 60–69, the prevalence of moderate-severe OSA was significantly higher in IPF patients, while the prevalence of mild OSA was similar, indicating that the presence of moderate-severe OSA is more likely to correlate with IPF pathogenesis.

The explanation of the relatively high prevalence of OSA among DPLD patients and the mechanistic interactions between them are not clear. As mentioned in previous studies, changes in lung volume may influence the patency of the upper airway and hence increase its collapsibility, known as the “tracheal tug” theory [29]. However, the association of lung function with AHI in DPLD patients is controversial. Mermigkis et al. found that AHI was negatively correlated with FEV1 [22], while Lancaster et al. reported no relationship between AHI and lung volume or DLCO [13]. Consistent with the latter, our study showed that there was no difference in FEV1%, FVC%, DLCO% or TLC% between OSA and non-OSA group. As temporality could not be established in this meta-analysis, a causal effect or relationship between OSA and progressive lung restriction is difficult to evaluate. Previous studies assumed that release of proinflammatory cytokines caused by underlying lung inflammation would also increase the likelihood of OSA. Sputum gene expression analyses have identified a significant increase for IL-6 and IL-8 in IPF patients [30]. Both IL-6 and IL-8 may mediate nasal inflammation in OSA patients, and were positively correlated with AHI [31]. Another possible explanation is that pulmonary function tests in identified studies were performed in an upright position but not in a supine position, which could not accurately reflect pulmonary function changes during sleep [13,23,26].

In our study, although the sleep architecture was not significantly altered in OSA group, the arousal index, ODI values and percentage of total sleep time with oxyhemoglobin saturation below 90% were much higher in patients with OSA, suggesting that OSA may impair sleep quality and exacerbate the nocturnal desaturation in DPLD patients. The presence of nocturnal desaturation, which even exceeded that of maximal exercise, was thought to be an independent predictor of worse survival after adjustment for age, gender, and BMI, and significantly correlated with pulmonary hypertension in patients with DPLD [10,32]. Nevertheless, daytime arterial oxygen tension was not influenced by comorbid OSA based on the pooled data, which is in accordance with previous studies showing only a loose correlation between nocturnal hypoxemia and daytime hypoxia (at rest or during exercise) [32,33]. Taken together, these findings highlight the need for early recognition of OSA and nocturnal oxygen therapy in DPLD patients. Recently, studies have shown that effective PAP treatment in IPF patients with OSA led to a significant improvement in the daily living activities, as well as quality of sleep and life, and may also influence the mortality in these patients [11,34].

To further explore the systematic impact of OSA on clinical outcomes in DPLDs, we evaluated whether OSA was associated with other comorbid conditions, such as cardiovascular and metabolic diseases. There was no significant difference among groups concerning hypertension, coronary artery disease, diabetes mellitus, GERD or stroke. However, the number of reports and the sample size was small to draw a conclusion that OSA may not increase the risk for other comorbid diseases in DPLDs. Further studies are thus warranted with sufficient power to examine this association.

Our study has several methodological limitations which should be taken into consideration when interpreting the results. First, most included studies were single-center with small sample size, and some clinical indicators were only evaluated in a few studies. For instance, some important lung function parameters or comorbidities were analyzed by pooling only five or four studies, which may weaken the effectiveness of meta-analysis and limit the generalizability of the findings. Second, as all the included studies had cross-sectional design, we could not evaluate or assume a causal effect or relationship between OSA and DPLDs. Third, although we use a random-effects model along with prespecified subgroup and sensitivity analyses to address the heterogeneity, our study was based on published literature, which limited the possibility to adjust potential confounders if they were not reported or not using unified data. High levels of undetected heterogeneity may exist in such small meta-analyses. Thus, pooled results of small subgroup analyses should be interpreted with caution and appropriate multivariate analysis will be necessary in future studies to examine the impact of OSA on DPLD outcomes, independent of other factors such as sex, age, type of DPLDs, disease severity, and treatment. Finally, publication and reporting bias were obvious in this meta-analysis when pooling the overall prevalence, which might be caused by a lack of publication of small studies with negative findings or an inflation of estimates by small studies [35,36]. However, previous evidence also demonstrates that publication bias may not affect the conclusions in most cases [37].

Despite the limitations, our study has several strengths. To the best of our knowledge, it is the first meta-analysis and systematic review on the prevalence and clinical impact of OSA in DPLDs, which compared the data among different types of DPLDs, offering evidence for decision-making in a real-world environment.

In conclusion, the present study reported an overall high prevalence of OSA in DPLDs, especially in IPF population, which may exacerbate the nocturnal desaturation and have negative influence on the outcomes. Larger studies with more homogeneous samples are warranted to better clarify the associations between OSA and different types of DPLDs, and to investigate potential clinical benefits of screening and managing OSA in DPLD patients.

## Supporting information

S1 Checklist PRISMA 2009 checklist. (DOC)

S1 Table Quality assessment for each study. (DOCX)

S2 Table Meta-analysis of the overall prevalence of OSA in DPLDs based on hypopnea scoring criteria. (DOCX)
