# Supplementary material for: Prevalence and impact of comorbid obstructive sleep apnoea in diffuse parenchymal lung diseases
Source: PLoS One. 2021 Feb 11;16(2):e0246878. doi: 10.1371/journal.pone.0246878 (PMC7877600; doi:10.1371/journal.pone.0246878)
Supplement: S1 Table — (DOCX) [file pone.0246878.s002.docx]

**Table S1. Quality assessment for each study**

| **Study** | **Prospective recruitment（0:No; 1:Yes）** | **Sample size justification (0: random sample;**  **1: consecutive sample)** | **Case definition (DPLD) (0: non-objective;**  **1: objective)** | **Sample representativeness (0: single-center; 1: multi-center; 2: recruited from general populations)** | **Description of inclusion criteria (0: No; 1: Yes)** | **Description of exclusion criteria (0: No; 1: Yes)** | **Outcome definition (OSA) (0:no specific criteria; l: specific criteria)** |
| --- | --- | --- | --- | --- | --- | --- | --- |
| Mermigki et al. 2007 | 1 | 1 | 1 | 0 | 0 | 0 | 1 |
| Lancaster et al. 2009 | 1 | 1 | 1 | 0 | 1 | 0 | 1 |
| Mermigki et al. 2010 | 1 | 1 | 1 | 1 | 0 | 0 | 1 |
| Kolilekas et al. 2013 | 1 | 1 | 1 | 0 | 0 | 0 | 1 |
| Pihtili et al. 2013 | 1 | 1 | 1 | 0 | 1 | 1 | 1 |
| Lee et al. 2015 | 1 | 1 | 1 | 0 | 1 | 1 | 1 |
| Bosi et al. 2017 | 1 | 1 | 1 | 0 | 0 | 1 | 1 |
| Gille et al. 2017 | 1 | 1 | 1 | 1 | 1 | 1 | 1 |
| Mavroudi et al. 2017 | 1 | 1 | 1 | 0 | 1 | 1 | 1 |
| Cardoso et al. 2018 | 1 | 1 | 0 | 0 | 1 | 1 | 1 |
| Zhang et al. 2019 | 1 | 1 | 1 | 0 | 0 | 1 | 1 |
| Troy et al. 2019 | 1 | 1 | 1 | 0 | 0 | 1 | 1 |
| Tudorache et al. 2019 | 0 | 1 | 1 | 0 | 0 | 1 | 1 |
| Sarac et al. 2019 | 1 | 1 | 1 | 0 | 1 | 1 | 1 |
| DPLD, diffuse parenchymal lung diseases; OSA, obstructive sleep apnoea. | | | | | | | |
