# Supplementary material for: Prevalence and impact of comorbid obstructive sleep apnoea in diffuse parenchymal lung diseases
Source: PLoS One. 2021 Feb 11;16(2):e0246878. doi: 10.1371/journal.pone.0246878 (PMC7877600; doi:10.1371/journal.pone.0246878)
Supplement: S2 Table — (DOCX) [file pone.0246878.s003.docx]

**Table S2. Meta-analysis of the overall prevalence of OSA in DPLDs based on hypopnea scoring criteria**

| **Variable** | **Number of**  **studies** | **Number of patients** | **Prevalence of OSA (95 % CI)** | **I^2^ (%)** |
| --- | --- | --- | --- | --- |
| Hypopnea scoring criteria |  |  |  |  |
| Oxygen desaturation of ≥3% | 6 ^[11, 17, 19, 22, 24, 26]^ | 307 | 0.72(0.62-0.82) | 76.3 |
| Oxygen desaturation of ≥4% | 3 ^[9, 18, 25]^ | 116 | 0.77(0.62-0.92) | 75.9 |
| Arousal and/or oxygen desaturation of ≥3% | 3 ^[12, 21, 23]^ | 160 | 0.73(0.55-0.91) | 84.9 |
| Arousal or an airflow reduction ≥ 30% with oxygen desaturation≥4% or an airflow reduction≥50% with an oxygen desaturation ≥ 3% | 1 ^[20]^ | 40 | 0.68(0.53-0.82) | - |
| DPLD, diffuse parenchymal lung diseases; OSA, obstructive sleep apnoea; CI, confidence interval. | | | | |
